# Exploring the neurobiological markers of suicidal behaviors in pediatric population: a narrative review

**DOI:** 10.3389/fped.2026.1738009

**Published:** 2026-05-28

**Authors:** Mona Salehi, Mahdieh Saeidi, Man Amanat, Tala Barias, Urenna Anyeji, Omar Alzein, Sasidhar Gunturu

**Affiliations:** 1 Department of Psychiatry, Bronx Care Health System, New York, NY, United States; 2 Department of Psychiatry, University of Minnesota School of Medicine, Minneapolis, MN, United States; 3 Department of Neurology, Mayo Clinic, Rochester, MN, United States; 4 Department of Psychiatry, Icahn School of Medicine at Mount Sinai, New York, NY, United States

**Keywords:** neurobiologic basis, pediatric, suicidal attempt, suicidal idea, suicide

## Abstract

**Background:**

Suicide is the second leading cause of death among children and adolescents, with rates of pediatric suicidal behavior rising substantially over the past two decades. The neurobiology of suicide has been extensively studied in adults, yet pediatric-specific evidence remains limited and the extent to which adult findings can be extrapolated to youth is unclear. This review synthesizes current evidence on the neurobiological correlates of suicidal ideation, suicide attempt, and death by suicide in pediatric and adolescent populations across neurological, genetic, epigenetic, inflammatory, metabolic, and endocrine domains.

**Methods:**

A literature search was conducted in PubMed, Embase, PsycINFO, and Google Scholar for peer-reviewed, English-language human studies. Priority was given to pediatric and adolescent samples, with adult data included where pediatric evidence was lacking. Studies were grouped by biological domain and by suicidal phenotype.

**Results:**

Suicidal ideation, suicide attempt, and death by suicide showed partially distinct biological signatures rather than lying on a single continuum of severity. Different markers, most notably cortisol regulation and stress-related DNA methylation, differed in direction between pediatric and adult cohorts, indicating that adult biomarker data cannot be directly extrapolated to youth. Findings converged on a developmental cascade in which genetic liability and early-life adversity influence the hypothalamic-pituitary-adrenal axis, with downstream effects on epigenetic regulation, neuroinflammation, neurochemistry, and frontolimbic circuitry.

**Conclusions:**

Pediatric suicidal behavior reflects developmentally distinct biological processes that cannot be inferred from adult findings. Advancing the field will require longitudinal, multimodal pediatric studies that disaggregate suicidal phenotypes, span the pubertal transition, and apply age-stratified reference ranges, supporting biologically informed stratification and mechanism-targeted intervention.

## Introduction

Suicide is defined as intentional self-inflicted harm resulting in death. It affects individuals across all age groups and is the second leading cause of death among youth and young adults, with rates rising by over 50% from 2000 to 2021 (1–4). Suicidal behaviors include ideation, attempts, and death by suicide (2).

Several studies have explored the role of environmental factors, such as low socioeconomic status, firearm availability, and strained parental relationships in elevating suicide risk (5–7). Although these findings suggest that environmental factors are significant, they are insufficient to fully explain suicidal behavior, particularly in youth. The rising rates of suicidality in this population underscore the need to investigate other contributing factors. Current research, however, is limited by small sample sizes, a primary focus on adult populations, and the difficulty of differentiating suicidal behavior from co-occurring mood disorders (8).

The role of biological mechanisms in suicidal ideation, attempt, and death by suicide is crucial yet understudied. As highlighted recently, gaps in understanding these processes contribute to suboptimal prevention outcomes and limited long-term interventions (9). Suicide is a highly complex and heterogeneous phenotype influenced by multifactorial psychological, environmental, neurological, and genetic risk factors. These dysregulate core neurobiological systems, including the serotonergic, endocannabinoid, and hypothalamic-pituitary-adrenal (HPA) axes. Additionally, early-life trauma impairs stress-coping capacity, promoting chronic inflammation that disrupts stress regulation, neuroplasticity, and neurotransmission (10).

Despite a growing literature on the neurobiology of suicide, important gaps remain. The great majority of mechanistic studies have been conducted in adult cohorts, and direct pediatric data are sparse across nearly every biological domain. Additionally, most studies pool suicidal ideation, suicide attempt, and death by suicide into a single outcome, obscuring phenotype-specific biological signatures. Lastly, individual biological domains have largely been studied in isolation, with few multimodal pediatric studies integrating neuroimaging, genomics, epigenetics, and peripheral biomarkers within the same cohort.

The objective of this narrative review is therefore to synthesize current evidence on the neurobiological correlates of suicidal ideation, suicide attempt, and death by suicide across six biological domains including neurological/neuroimaging, genetic, epigenetic, inflammatory and immune, metabolic, and endocrine systems with priority given to pediatric and adolescent samples; to highlight findings in which pediatric biology diverges from adult biology; and to propose a developmentally informed integrative framework to guide future research and translational efforts. Throughout the review, evidence is organized by suicidal phenotype wherever the underlying studies permit, in order to clarify domain-specific and phenotype-specific patterns.

## Methods

### Information sources and eligibility criteria

A literature search was conducted in PubMed, Embase, PsycINFO, and Google Scholar to identify relevant studies on the neurobiological correlates of suicidal behavior in pediatric populations. Inclusion criteria were: (i) peer-reviewed human studies; (ii) English language; (iii) studies reporting on at least one neurobiological correlate (neuroimaging, neurotransmitter, genetic, epigenetic, immune/inflammatory, metabolic, or endocrine) of suicidal ideation, suicide attempt, or death by suicide; and (iv) studies in which the suicidal phenotype was explicitly defined, with priority given to publications from the past two decades. Non-peer-reviewed sources, case reports without biological measurement, and animal-only studies were excluded.

### Search terms

Search terms combined keywords related to the suicidal phenotype (“*suicidal ideation,” “suicide attempt,” “suicidal behavior,” “death by suicide,” “self-harm,” “adolescent suicide,” “pediatric suicide”*) with terms reflecting the biological domains reviewed (“*neuroimaging,” “MRI,” “fMRI,” “neurocircuitry,” “serotonin,” “neurotransmitter,” “BDNF,” “GWAS,” “DNA methylation,” “epigenetic,” “interleukin,” “IL-6,” “cytokine,” “CRP,” “metabolic syndrome,” “HPA axis,” “cortisol,” “leptin”*). Boolean operators (AND, OR) were used to combine concepts. Reference lists of retrieved articles, relevant systematic reviews, and meta-analyses were also screened to identify additional eligible studies. Priority was given to studies including pediatric or adolescent samples (≤18 years); adult studies were included where pediatric data were unavailable but the findings were considered relevant to developmental neurobiology.

### Synthesis approach

Evidence was synthesized qualitatively. For each biological domain, the most reproducible findings, the principal areas of inconsistency, and the available pediatric-specific evidence are summarized. Quantitative pooling was not performed given the heterogeneity of designs, samples, and outcomes; however, where large meta-analyses or population-based cohorts existed, their effect estimates are reported directly. Findings across domains were then integrated into a developmental cascade model centered on the hypothalamic-pituitary-adrenal (HPA) axis (Integrative Framework).

## Results

### Neurological factors

Neuroimaging research has provided valuable insight into the brain mechanisms underlying suicidal thoughts and behaviors in youth (11). These studies consistently implicate neural circuits involved in emotion regulation, impulse control, and decision-making. These domains are often disrupted in individuals at risk for suicide. Although findings vary across studies, several key neuroanatomical and functional patterns have emerged.

### Structural brain differences

Structural MRI studies suggest that suicidal youth often exhibit subtle but significant alterations in regions responsible for emotional regulation and executive functioning. Reduced gray matter volume in the orbitofrontal cortex (OFC), anterior cingulate cortex (ACC), hippocampus, and amygdala has been linked to suicidal ideation and attempts (12, 13). These regions form part of the frontolimbic circuit, which coordinates emotional responses and behavioral inhibition. Disruption in this circuit may impair the ability to manage distressing emotions, contributing to impulsive or self-destructive behavior.

In one large pediatric study from the Adolescent Brain Cognitive Development (ABCD) cohort (14), children aged 9–10 with suicidal thoughts and behaviors showed minimal macroscopic structural differences, aside from a thinner left superior temporal sulcus. This suggests that significant neuroanatomical changes may emerge later in adolescence as brain networks mature. A study on pediatric population aged from 11 to 18 years old has reported smaller surface area in prefrontal, temporal, and parietal cortices among suicide attempters, with atypical developmental trajectories showing thicker cortices over time, possibly reflecting delayed cortical pruning (13). These findings suggest that atypical cortical thickening in youth with suicidal behavior may reflect delayed or incomplete synaptic pruning during adolescence. Such maturational lag within prefrontal and temporal networks could impair emotional regulation, impulse control, and stress response which are core domains implicated in vulnerability to suicidal behavior.

### Functional brain alterations

Functional MRI (fMRI) studies further demonstrate abnormal activation patterns in frontolimbic and temporal circuits. For instance, adolescents with a history of suicide attempts exhibit reduced activity in prefrontal regions which are involved in self-control and decision-making, and heightened activity in temporal and limbic areas linked to emotional reactivity (15, 16). This imbalance may predispose youth to impulsive actions under emotional stress.

One measure often used in resting-state fMRI is the amplitude of low-frequency fluctuations (ALFF), which reflects spontaneous neural activity. In a study by *Cao* et al. (15), suicidal adolescents displayed increased ALFF in the temporal and occipital cortices but decreased ALFF in the superior and middle frontal gyri. These findings suggest excessive baseline activation in emotional and perceptual areas, coupled with underactivity in cognitive control regions. Of note, these measures could distinguish suicidal from non-suicidal depressed youth with over 80% sensitivity, indicating potential biomarker value.

### Network connectivity and the default mode network

Resting-state studies have also examined alterations in the default mode network (DMN)—a set of brain regions active during rest and self-referential thinking, including the posterior cingulate cortex (PCC), precuneus, and medial prefrontal cortex. The DMN is crucial for processes like introspection, future planning, and evaluating one's sense of self. Abnormal connectivity within this network may underlie maladaptive self-focus and rumination, both of which are common in suicidal individuals.

One sex- and age-matched case-control study found that suicidal adolescents with depression showed reduced DMN connectivity in the PCC and increased connectivity in the left cerebellum, suggesting altered coordination between self-referential and emotional processing regions (17). Similarly, a study on adolescent females reported enhanced connectivity between the insula, cingulate cortex, and prefrontal areas, which may reflect heightened emotional awareness but poor regulatory control (16). Table 1 summarizes the principal neuroimaging studies discussed in this section, including sample characteristics, imaging modality, brain regions implicated, and the suicidal phenotype assessed in each study.

**Table 1:** Neuroimaging findings in pediatric and adolescent suicidality.

Study (Year, Ref)	Sample (n; age)	Modality	Region/Network	Key Finding	Phenotype
Schmaal et al., 2019 (11)	Narrative review of 2 decades of neuroimaging studies; mixed adult and adolescent samples	Structural, functional, and molecular neuroimaging (review)	VPFC, DPFC, insula, mesial-temporal, striatal, and posterior connections	Convergent alterations in emotion- and impulse-regulation circuits. VPFC alterations linked to ideation; DPFC and inferior frontal gyrus alterations linked to attempt.	Ideation and attempt
Johnston et al., 2017 (12)	26 BD attempters+42 BD non-attempters+45 HC; ages 14–25 y	Multimodal: sMRI, DTI, task-based fMRI	Ventral frontolimbic system; amygdala–PFC connectivity	↓ gray matter, ↓ white matter integrity, ↓ amygdala–PFC connectivity in attempters. Reductions correlated with ideation severity and attempt lethality.	Attempt
Gifuni et al., 2021 (13)	28 MDD attempters+34 MDD non-attempters+30 HC adolescents; mean age 15.9 y, range 11.6–18.1 y	sMRI (cortical surface area and thickness)	Prefrontal, temporal, and parietal cortices	Smaller cortical surface area in attempters; atypical thickening trajectory consistent with delayed synaptic pruning.	Attempt
Vidal-Ribas et al., 2021 (14)	ABCD cohort (*N* = 7,994); ages 9–10 y; population-based	Multimodal sMRI+rs-fMRI+task-based fMRI	Whole-brain; left superior temporal sulcus	Minimal macroscopic differences overall; only thinner left superior temporal sulcus distinguished children with STB. Suggests neurobiological differences emerge later in adolescence.	Ideation and attempt
Cao et al., 2016 (15)	Young patients with MDD ± suicidal behavior; ages 15–29 y	rs-fMRI (ALFF)	Right superior, left middle temporal, left middle occipital, superior and middle frontal gyri	↑ ALFF in temporal and occipital cortices and ↓ ALFF in superior and middle frontal gyri in suicidal vs. non-suicidal depressed patients.	Mixed (ideation and attempt)
Liu et al., 2022 (16)	31 female MDD attempters+27 MDD non-attempters+36 HC; ages 12–18 y	rs-fMRI (ALFF, fALFF, ReHo, functional connectivity)	Insula, cingulate cortex, prefrontal regions (frontolimbic circuit)	↑ connectivity between insula, cingulate cortex, and prefrontal areas in attempters.	Attempt
Zhang et al., 2016 (17)	35 suicidal depressed adolescents+18 non-suicidal depressed+47 HC; ages 15–29 y	rs-fMRI (independent component analysis)	Default mode network (PCC, precuneus, mPFC); left cerebellum	↓ DMN connectivity in PCC and ↑ connectivity in left cerebellum in suicidal adolescents.	Mixed (ideation and attempt)

ABCD, adolescent brain cognitive development; ACC, anterior cingulate cortex; ALFF, amplitude of low-frequency fluctuations; DMN, default mode network; DPFC, dorsal prefrontal cortex; DTI, diffusion tensor imaging; fMRI, functional magnetic resonance imaging; IFG, inferior frontal gyrus; mPFC, medial prefrontal cortex; OFC, orbitofrontal cortex; PCC, posterior cingulate cortex; PFC, prefrontal cortex; sMRI, structural magnetic resonance imaging; STB, suicidal thoughts and behaviors; VPFC, ventral prefrontal cortex.

### Neurotransmitter system

Neurotransmitter dysregulation plays a central role in the pathophysiology of suicidal behavior. Several studies have reported that suicidal behavior arises from a complex interplay between monoaminergic, neurotrophic, and excitatory–inhibitory systems, with serotonergic dysfunction representing a core neurochemical substrate (18, 19). Lower central serotonin (5-HT) activity has been associated with increased suicide risk, independent of psychiatric diagnoses (18). The prefrontal cortex (PFC), particularly its ventral and medial regions, exerts top-down modulation of serotonergic neurons in the dorsal and median raphe nuclei, thereby influencing emotional regulation, patience, and cognitive flexibility (20–22). Disruptions in serotonergic signaling may heighten vulnerability to suicidality.

Biochemical studies have shown reduced cerebrospinal fluid (CSF) concentrations of 5-hydroxyindoleacetic acid (5-HIAA), the main serotonin metabolite, among suicide attempters, especially in those employing violent methods (23, 24). Postmortem and imaging findings show decreased serotonin transporter (SERT) binding in the ventral PFC of suicide victims, suggesting region-specific serotonergic deficits linked to behavioral dyscontrol (25). In adolescents, peripheral markers of serotonin function also appear altered. Lower plasma and whole-blood serotonin levels, reduced platelet aggregation responses, and diminished tryptophan concentrations have all been observed in suicidal youth (26, 27). These findings suggest that serotonergic abnormalities extend beyond central pathways and may reflect systemic dysregulation of serotonin metabolism.

Neurotrophic mechanisms further intersect with these changes. Decreased expression of brain-derived neurotrophic factor (BDNF) and its receptor TrkB in the PFC and hippocampus of teenage suicide victims indicates impaired neuroplasticity and stress adaptation (28). This aligns with preclinical evidence showing that serotonergic deficits and reduced BDNF signaling converge to limit synaptic remodeling and emotional resilience under chronic stress.

Abnormalities in dopaminergic, glutamatergic, GABAergic, and endocannabinoid systems have also been implicated in suicide. Lower CSF homovanillic acid (HVA) levels in suicide attempters point to low dopaminergic function, possibly underlying anhedonia and motivational deficits (29, 30). Dysregulated glutamate and GABA gene expression in the PFC suggest an imbalance between excitatory and inhibitory neurotransmission (31). Alterations in CB1 receptor expression in the dorsolateral prefrontal cortex (PFC) of suicide victims, particularly among those with comorbid substance use, further implicate the endocannabinoid system in emotional regulation and stress response (32–34).

### Genetic contributors

Suicidal thoughts and behaviors have a significant genetic component, with heritability estimates ranging from 30% to 55% in twin and family studies (35). These findings indicate that genetic factors contribute meaningfully to suicide risk, though the effect is polygenic and driven by many small-effect variants rather than single-gene mutations (36). Genetic predisposition often overlaps with psychiatric disorders such as major depressive disorder (MDD), bipolar disorder, and schizophrenia, yet increasing evidence suggests that suicidal behaviors can arise from genetic influences partly independent of these conditions (37).

### Genome-wide association studies (GWAS)

GWAS systematically scan millions of single-nucleotide polymorphisms (SNPs) across the genome to identify variants associated with suicidal phenotypes. Large-scale GWAS from the Psychiatric Genomics Consortium (PGC) and UK Biobank have identified several risk loci associated with suicidal attempt. For instance, *Docherty* et al. analyzed over 44,000 cases and 900,000 controls, identifying 12 risk loci near *DRD2, SLC6A9, FURIN, NLGN1, SOX5, PDE4B, and CACNG2* (38). The CACNG2 gene, encoding voltage-gated calcium channel, has repeatedly been implicated in mood regulation and impulsivity across cohorts (39). The *NLGN1* (neuroligin 1) gene encodes a member of a family of neuronal cell surface proteins involved in synaptogenesis, and variants were linked to suicidal behavior in another large-scale GWAS (40).

Although GWAS identify population-level associations, Polygenic Risk Scores (PRS) quantify the cumulative inherited risk in individuals. PRS are calculated by summing risk alleles weighted by their GWAS-derived effect sizes, thereby estimating a person's overall genetic liability for a given trait. *Ruderfer* et al. found that PRS for depressive symptoms, neuroticism, major depressive disorder, and schizophrenia predicted suicide attempts in about 160,000 participants (41). Additionally, some studies suggest bipolar disorder PRS may correlate with suicide attempts, though effect sizes and adjustment for covariates vary and no robust large-scale estimate has been consistently replicated (42) PRS for ADHD and autism spectrum disorder (ASD) also showed positive associations with suicidal ideation in children and adolescents (43).

### Candidate-gene approaches

Although largely complemented by GWAS, candidate-gene investigations have provided critical early insight into molecular pathways contributing to suicidal behavior. These studies have focused on genes involved in stress regulation, monoaminergic neurotransmission, and neurotrophic signaling, which are biologically plausible mechanisms linking environmental adversity to suicidal risk. Variants in tryptophan hydroxylase (TPH1, TPH2), serotonin transporter (5-HTTLPR), and serotonin receptors (5-HTR1A, 5-HTR2A) have been associated with suicidal behaviors in adults, though replication in pediatric cohorts remains limited (44, 45). The most studied genes are presented in Table 2.

**Table 2:** Stress-Related genes implicated in suicidal behavior.

gene	protein/function	key polymorphisms	molecular/physiological effect	expression & trauma interaction	relevance to suicidal behavior
NR3C1	Glucocorticoid Receptor (GCR); mediates cortisol feedback in HPA axis	rs6198, rs41423247, rs6191	Alters GCR sensitivity → impaired cortisol feedback, increased HPA axis activity	Overexpressed in suicide attempters with high physical abuse, sexual abuse, and emotional neglect (46–49).	Heightened stress reactivity, mood dysregulation, increased suicidal vulnerability
NR3C2	Mineralocorticoid Receptor (MR); regulates basal cortisol tone and stress recovery	rs5522	Modulates MR activity → affects sodium balance, HPA axis initiation	Underexpressed in trauma-exposed SA patients (*p* < 0.0001), except physical neglect. Underexpression in rs5522-G carriers (47).	Weakened stress recovery, prolonged HPA activation, emotional dysregulation, suicide risk
FKBP5	Co-chaperone regulating GCR sensitivity	rs1360780, rs3800373, rs9470080	Decreases GCR sensitivity → prolonged cortisol release, impaired negative feedback	Risk alleles+childhood trauma → increased FKBP5 expression, glucocorticoid resistance (50)	Increased suicidal ideation and attempts in trauma-exposed carriers
SLC6A4	Serotonin transporter (5-HTT)	5-HTTLPR (S and L alleles)	S allele reduces transcription → decreased serotonin reuptake	S allele carriers show increased stress sensitivity and amygdala reactivity (51–53)	Higher odds of suicide attempts under psychosocial stress

### Epigenetic mechanisms

Epigenetic mechanisms represent the dynamic layer through which environmental stressors alter gene expression without modifying DNA sequence. DNA methylation, histone modification, and non-coding RNA regulation mediate enduring transcriptional changes in stress-related neural circuits. The most extensively studied mechanism is DNA methylation, in which methyl groups added to cytosine residues within CpG sites modulate transcriptional activity (54). Early-life adversity, trauma, and chronic stress can induce stable methylation changes in stress-responsive genes, effectively embedding environmental experiences into the genome's regulatory architecture.

### DNA methylation and the stress response

One of the most consistent findings in suicide research is hypermethylation of specific CpG sites in the NR3C1 promoter region, particularly exon 1F, which reduces glucocorticoid receptor expression and disrupts negative feedback within the HPA axis (55). *McGowan* et al. first demonstrated this in post-mortem hippocampal samples of suicide victims with a history of childhood abuse, showing methylation levels approximately 2-fold higher at key CpG sites than in controls (55). Subsequent studies have confirmed similar patterns in peripheral blood, suggesting a promising but not yet clinically validated peripheral biomarker of central stress dysregulation (56, 57).

Methylation alterations in corticotropin-releasing hormone (*CRH*), spindle and kinetochore-associated complex subunit 2 (*SKA2*), brain-derived neurotrophic factor (*BDNF*), and *SLC6A4* have also been implicated in suicidal behavior. *Jokinen* et al. identified two *CRH* promoter CpG sites (*cg19035496* and *cg23409074*) that were hypomethylated in adults with violent suicide attempts but hypermethylated in high-risk adolescents, suggesting age-dependent differences in stress regulation (58). *SKA2*, involved in glucocorticoid receptor nuclear translocation, shows lower expression and increased promoter methylation in individuals who died by suicide; these patterns correlate with elevated waking cortisol and suicidal ideation (59). Likewise, methylation changes in *BDNF* and *SLC6A4* promoters have been linked to diminished neurotrophic support and altered serotonergic tone, respectively, both correlating with suicidal ideation (60, 61).

Recent large-scale epigenome-wide association studies (EWAS) have begun to identify differential methylation sites across the genome, implicating pathways involved in immune activation, neuronal development, and circadian regulation (62). However, the specificity of these marks to suicidality remains uncertain, as many also occur in stress-related or inflammatory conditions.

Of note, global methylation measures are unable to capture stress-related risk. *Perret* et al. examined whether childhood peer victimization was reflected in established epigenetic aging indices such as the Horvath1/2 clocks, DunedinPACE, and Epistress glucocorticoid scores. Although victimized youth exhibited higher rates of later depression and suicidality, these broad methylation-age metrics did not differ between exposed and control groups (63). These findings demonstrate that such global indices primarily track biological aging rather than specific stress-responsive methylation alterations, and underscore the need for more targeted epigenetic biomarkers reflecting cumulative psychosocial stress rather than general methylation drift (63).

### Inflammatory and immune mechanisms

Growing evidence implicates immune dysregulation and inflammatory signaling in the biological vulnerability underlying suicidal ideation, suicide attempt, and death by suicide. The current findings are, however, not uniform across cytokines. Among inflammatory markers, interleukin-6 (IL-6) shows the most consistent association with suicidality. Elevated IL-6 levels have been reported in serum, cerebrospinal fluid (CSF), and post-mortem prefrontal cortex of individuals with suicidal ideation or attempts (64–68). In a cohort of 63 suicide attempters, CSF IL-6 levels were highest among violent suicide attempters, and correlated with monoamine metabolites 5-HIAA and HVA, suggesting interactions between inflammatory signaling and serotonergic/dopaminergic neurotransmission (69). Another study found higher IL-6 levels correlated with the lethality of suicidal attempts, further highlighting its potential as a state-dependent biomarker (70).

In contrast, results for other inflammatory cytokines including interleukin-1β (IL-1β) and tumor necrosis factor-α (TNF-α) are not consistent across studies. Some investigations report increased levels in serum or post-mortem PFC, while others find no significant differences between suicidal individuals and psychiatric or healthy controls (71). Anti-inflammatory cytokines demonstrate similar variability. IL-2 levels have been reported as reduced in some studies of suicide attempters (72, 73), yet others find no significant change (Table 3). Findings on IL-4, TGF-*β*, and other regulatory cytokines are further inconsistent (71).

**Table 3:** Inflammatory and immune markers in suicidal behavior.

Marker	Direction of change	Sample type	Key studies (year, ref)	Pediatric/adolescent evidence	Phenotype
IL-6	↑ Elevated	Serum, plasma, CSF, post-mortem PFC	Lindqvist 2009 (CSF; *n* = 63 attempters+47 HC, adults; highest in violent attempters; correlated with 5-HIAA, HVA). Pandey 2012 (post-mortem PFC; *n* = 24 teen suicide victims+24 HC; ↑ mRNA & protein in BA-10).	Direct pediatric evidence: Pandey 2012 in teenage suicide victims (post-mortem). Most CSF/serum data in adults; pediatric replication limited.	Attempt; death by suicide (post-mortem)
IL-1β	↑ Elevated (post-mortem); inconsistent in serum	Post-mortem PFC; serum (variable)	Pandey 2012 (post-mortem PFC; *n* = 24 teen suicide victims+24 HC; ↑ mRNA & protein in BA-10). Serum studies show inconsistent direction.	Direct pediatric evidence in post-mortem brain (Pandey 2012). Peripheral data in adolescents lacking and inconsistent.	Death by suicide (post-mortem); inconsistent for ideation/attempt
TNF-α	↑ Elevated (post-mortem); inconsistent in serum	Post-mortem PFC; serum (variable)	Pandey 2012 (post-mortem PFC; *n* = 24 teen suicide victims+24 HC; ↑ mRNA & protein in BA-10). Adult serum data inconsistent across studies.	Direct pediatric post-mortem data (Pandey 2012). Some adolescent peripheral studies report decreased TNF-α, conflicting with adult literature.	Death by suicide (post-mortem); inconsistent otherwise
IL-2	↓ Reduced (some studies); no change (others)	Serum	Several adult cohorts of suicide attempters; results not consistently replicated.	Pediatric data limited; primarily adult studies.	Attempt (mostly adult)
IL-4, TGF-β	Variable	Serum, plasma; some post-mortem	Anti-inflammatory cytokines; results inconsistent across studies and populations.	Pediatric data limited.	Mixed phenotypes (mostly adult)
CRP	↑ Elevated	Serum	Multiple adult cohorts of suicidal individuals show elevated CRP vs. psychiatric and healthy controls.	Adolescent data emerging but limited; consistent direction with adult findings.	Ideation and attempt
Fibrinogen	↑ Elevated	Serum	Established in adult psychoneuroimmunology; consistent with broader inflammatory profile.	Pediatric data sparse.	Ideation and attempt (adult)
TRYCATs (kynurenine pathway)	↑ Elevated	Serum, plasma, CSF	Activation of TRYCAT pathway diverts tryptophan from serotonin synthesis toward neurotoxic kynurenine metabolites (e.g., quinolinic acid; NMDA-receptor agonist).	Mechanistic data from adults; pediatric replication needed.	Attempt (mostly adult)
Omega-3 PUFAs (EPA, DHA)	↓ Reduced	Plasma	Prospective cohort of MDD patients who later attempted suicide showed lower plasma EPA/DHA. Anti-inflammatory and neuroprotective lipids.	Pediatric studies limited.	Attempt

CRP, C-reactive protein; CSF, cerebrospinal fluid; DHA, docosahexaenoic acid; EPA, eicosapentaenoic acid; IL, interleukin; MDD, major depressive disorder; NMDA, N-methyl-D-aspartate; PFC, prefrontal cortex; PUFA, polyunsaturated fatty acid; TGF-β, transforming growth factor beta; TNF-α, tumor necrosis factor alpha; TRYCATs, tryptophan catabolites.

The inconsistencies likely stem from small sample sizes, cross-sectional study designs, differences in biological sample type (serum vs. plasma vs. CSF), time from attempt/death to sample collection, psychiatric diagnosis heterogeneity, medication status, and assay variability. To resolve these discrepancies in cytokine results, future studies should adopt longitudinal, multi-center designs with larger cohorts, standardized cytokine measurement protocols (including synchronized serum and CSF collection), and stratification based on age, sex, psychiatric diagnosis, medication status, violent vs. non-violent attempt, and timing relative to suicidal behavior. Integration of inflammatory markers with neuroimaging, genomic/epigenetic data, and multi-omics profiling may help identify biologically distinct subtypes of suicidal patients and improve biomarker reliability.

Early psychoneuroimmunology research established that peripheral inflammatory activity reflected in elevated C-reactive protein (CRP), fibrinogen, and tryptophan catabolites (TRYCATs) is higher among suicidal individuals than in psychiatric or healthy controls (74–76). Of note, activation of the TRYCAT pathway diverts tryptophan metabolism away from serotonin synthesis, generating neurotoxic metabolites such as quinolinic acid that can overstimulate N-methyl-D-aspartate (NMDA) receptors and exacerbate mood dysregulation (77).

Alterations in lipid metabolism and oxidative stress further link inflammation to suicide risk. Prospective data demonstrate that individuals with major depressive disorder who later attempt suicide exhibit significantly lower plasma levels of omega-3 polyunsaturated fatty acids (PUFAs), particularly eicosapentaenoic acid (EPA) and docosahexaenoic acid (DHA) (78). These fatty acids possess strong anti-inflammatory and neuroprotective properties, and their deficiency may reduce membrane fluidity, impair serotonergic signaling, and amplify oxidative stress, thereby heightening suicide vulnerability (79).

The association between immune dysfunction and suicidality appears particularly strong in individuals exposed to early-life adversity. Childhood maltreatment induces lasting low-grade inflammation through persistent activation of microglia and peripheral immune cells, resulting in increased release of pro-inflammatory cytokines and nitric oxide, alongside reduced neurotrophic support (80). This process can lead to maladaptive programming of the HPA axis, creating a lifelong hyper-reactive stress system. Meta-analyses indicate that adults with a history of childhood trauma maintain elevated CRP and IL-6 levels decades later, correlating with increased suicidal ideation and attempt rates (81). Such findings support a model of neuro-immune sensitization, in which early inflammatory priming of stress and affective circuits fosters enduring vulnerability to suicidality (82).

### Metabolic risk factors

Multiple epidemiological and clinical studies have reported an association between metabolic syndrome and suicidal attempts (83–85), but the directionality and causality of this relationship remain unresolved. It is unclear whether metabolic abnormalities act as causal drivers of suicidality, represent downstream consequences of psychiatric illness or its treatments, or simply constitute markers of shared pathophysiology such as chronic inflammation, poor diet, or sedentary lifestyle. A recent longitudinal, population-based study of more than 380,000 participants found that the presence of metabolic syndrome at baseline was associated with an increased risk of subsequent suicide events after matching for age, sex, educational attainment, smoking status, household income, and psychiatric history (schizophrenia, bipolar disorder, and major depressive disorder) (86). These findings also showed a dose–response relationship with the number of metabolic syndrome components present at baseline correlated positively with the risk of suicide attempts resulting in hospitalization or death, strengthening the epidemiological case for a graded relationship between metabolic burden and severe suicidal outcomes (86).

Despite these robust associations, causality remains unestablished. This UK Biobank study (86) provided evidence of bidirectional mediation between cognitive performance and metabolic syndrome in predicting future suicide risk. Metabolic dysfunction was associated with declines in cognitive function that subsequently increased suicide risk, while baseline cognitive deficits predicted behaviors that worsened metabolic health. Such bidirectional effects are compatible with both causal and non-causal models (86). Alternative explanations include reverse causation, in which severe mood disorders, maladaptive behaviors, or psychotropic medication side effects drive metabolic disturbances. The association may also be confounded by systemic inflammation, which promotes both metabolic dysregulation and neuropsychiatric vulnerability (87). A biologically plausible shared antecedent is diminished central serotonergic function. Reduced serotonin availability has been linked to increased appetite and weight gain, central adiposity, impaired glucose tolerance, and insulin resistance, alongside depressed mood (88).

### Endocrine risk factors

Endocrine abnormalities have been studied as potential contributors to suicidal behavior, particularly through dysregulation of the HPA axis and stress-response systems. However, results remain heterogeneous.

Cortisol is one of the most extensively studied endocrine biomarkers in relation to suicidal behavior. A large meta-analysis including 30 studies with over 1,700 individuals with suicidal behavior and 2,000 controls reported that individuals with suicidal ideation or attempts had higher overall cortisol levels than healthy controls (89). However, when morning cortisol was examined specifically, levels were paradoxically lower in suicidal individuals relative to healthy controls, highlighting the importance of circadian timing in hormonal assessment. This finding reflects a dysregulated adaptation of the HPA axis following chronic stress exposure. Under sustained psychological stress, repeated hyperactivation of the HPA axis may lead to compensatory downregulation of corticotropin-releasing hormone (CRH) neurons, reduced adrenal sensitivity, or glucocorticoid receptor resistance, ultimately resulting in a blunted cortisol awakening response. Additionally, when suicidal individuals were compared to psychiatric controls rather than healthy individuals, cortisol levels were significantly lower. This suggests that hypercortisolemia may reflect general psychological distress, whereas reduced cortisol within psychiatric populations may represent chronic HPA axis exhaustion or glucocorticoid receptor desensitization.

A second meta-analysis of 27 studies involving over 2,000 participants found age significantly modified the cortisol and suicide relationship. In samples with a mean age below 40 years, individuals with a history of suicide attempts exhibited higher cortisol levels, whereas studies with mean ages above 40 showed lower cortisol in suicidal individuals compared to controls (90). This age-dependent reversal is especially relevant to adolescents, whose stress-response systems are still maturing. During adolescence, the HPA axis is more reactive to stress and more sensitive to social threat, peer rejection, and early trauma. Therefore, many adolescent suicide attempters demonstrate hypercortisolism, rather than the hypocortisolism more commonly observed in adults.

The interpretation of cortisol findings in suicidal individuals must be approached with caution due to the methodological heterogeneity. Cortisol secretion follows a diurnal pattern with a sharp increase shortly after awakening and gradual decline throughout the day. Differences in biological specimen type also affect interpretation; plasma and saliva reflect acute changes, whereas CSF and hair cortisol capture longer-term HPA axis activity. In addition, medication exposure, duration of illness, comorbid anxiety or substance use, sleep disruption, and menstrual or pubertal hormonal fluctuations can alter glucocorticoid signaling. Acute suicidal crises may be characterized by heightened cortisol and CRH activity, whereas repeated self-harm and chronic stress may lead to reduced cortisol through downregulation of glucocorticoid receptors and impaired feedback inhibition.

Studies have also investigated additional endocrine systems beyond the HPA axis, examining the potential roles of thyroid hormones, sex hormones, prolactin, oxytocin, and leptin in mood disorders and suicidal behavior. Several studies report lower circulating leptin levels in suicidal individuals (91) A genetic study in adolescents found that carriers of the mutant allele in the *rs1171276* region of the leptin gene were more likely to exhibit suicidal behavior (92). Findings from other endocrine systems, although heterogeneous and not always consistent, offer further insight into the complex neuroendocrine alterations associated with these conditions (93).

## Discussion

Several themes emerge from the evidence reviewed above. Suicidal ideation, suicide attempt, and death by suicide show partially distinct biological signatures rather than lying on a single continuum, and the biology of suicide risk in youth differs from that in adults, with some markers reversing direction across the lifespan. At the same time, many findings are reproducible in direction but inconsistent in magnitude, and a smaller number are inconsistent even in direction. These inconsistencies are best understood as reflecting the structure of the evidence base. Sample sizes are typically small, and many candidate-gene and cytokine studies in suicide are underpowered to detect the modest effect sizes observed in larger consortia. Study designs are predominantly cross-sectional, conflating state-related signals proximal to a suicidal episode with trait-related signals reflecting stable vulnerability. Biological sample type also varies considerably across studies, with serum, plasma, cerebrospinal fluid, post-mortem brain tissue, and saliva capturing different physiological compartments and limiting direct comparability. Demographic and clinical heterogeneity, including age, sex, pubertal stage, psychiatric diagnosis, medication exposure, and time from suicidal event to biological sampling, has rarely been controlled in a uniform manner.

### Phenotype-specific patterns

When organized by the suicidal phenotype assessed in each underlying study (Table 4), the evidence reviewed in this manuscript reveals partially distinct biological signatures across suicidal ideation, suicide attempt, and death by suicide. Genetic studies provide the clearest demonstration of this distinction. Using Swedish national registry data on more than a million individuals, *Edwards* et al. (6) estimated how much of the genetic risk for suicide attempt overlaps with the genetic risk for death by suicide. They found substantial overlap (genetic correlation of 0.67 in women and 0.74 in men) but the overlap was incomplete, with a substantial proportion of genetic risk is unique to each phenotype. Importantly, the data did not fit a model in which attempt and death sit on a single continuum of severity, suggesting that these outcomes are not simply mild and severe versions of the same biological process (6). At the molecular level, the largest genome-wide meta-analysis of suicide attempt identified twelve genome-wide significant loci across about 44,000 cases (38), while a separate meta-analysis of suicide death implicated *NLGN1*, with the association persisting after conditioning on attempt status (40). Phenotype-specific patterns also emerge in pediatric neuroimaging. A systematic review of 28 adolescent magnetic resonance imaging studies recently proposed the Emotional Pain and Social Disconnect (END) model, in which a circuit comprising the cerebellum, amygdala, and hippocampus is altered in adolescents with suicidal ideation and more strongly altered in those with a history of attempt, whereas a separate circuit comprising the lateral orbitofrontal cortex and temporal gyri appears specific to adolescent attempters (94). Together, these findings argue against treating suicidal behavior as a unitary biological entity and support phenotype-stratified study designs in future pediatric research.

**Table 4:** Evidence by suicidal phenotype: suicidal ideation, suicide attempt, and death by suicide.

Biological domain/marker	Suicidal ideation	Suicide attempt	Death by suicide	Manuscript refs
Fronto-limbic structure & connectivity	Reduced gray matter volume in OFC, ACC, hippocampus, and amygdala (linked to both ideation and attempts). Ventral PFC alterations particularly associated with ideation. Reduced posterior cingulate cortex connectivity within the default mode network in suicidal depressed adolescents. Amygdala–prefrontal functional connectivity reductions correlate with ideation severity.	Reduced gray matter volume in frontolimbic regions (linked to both ideation and attempts). Dorsal PFC and inferior frontal gyrus abnormalities particularly associated with attempt behaviors. Smaller cortical surface area in adolescent attempters with atypical thickening over time, suggesting delayed pruning. Reduced ventral frontolimbic connectivity correlates with attempt lethality.	Reduced SERT binding in the ventral PFC of suicide victims (post-mortem imaging and tissue). Reduced BDNF and TrkB expression in the PFC and hippocampus of teenage suicide victims. Elevated pro-inflammatory cytokines (IL-1β, IL-6, TNF-α) in the post-mortem prefrontal cortex of teenage suicide victims.	11, 12, 13, 17, 25, 28, 67
Serotonergic system	Methylation changes in the SLC6A4 (serotonin transporter) promoter linked to altered serotonergic tone, correlating with suicidal ideation. Altered peripheral serotonergic markers (lower plasma and whole-blood serotonin, reduced platelet aggregation, diminished tryptophan) reported in suicidal youth, encompassing both ideation and attempts.	Reduced cerebrospinal fluid 5-HIAA in suicide attempters, particularly in those employing violent methods. Altered peripheral serotonergic markers also reported in suicidal youth.	Reduced SERT binding in the ventral PFC of suicide victims (post-mortem). Reduced BDNF and TrkB expression in the PFC and hippocampus of teenage suicide victims, indicating impaired neuroplasticity and stress adaptation.	23, 25, 26, 27, 28, 61
HPA axis/cortisol	SKA2 hypomethylation correlates with both elevated waking cortisol and suicidal ideation. CRH promoter methylation alterations identified in high-risk adolescent cohorts.	Higher cortisol levels in suicide attempters when sample mean age is below 40 years; the direction reverses to lower cortisol in samples above 40 years (age-dependent effect particularly relevant to pediatric populations). Two CRH promoter CpG sites are hypomethylated in adults with violent suicide attempts.	NR3C1 exon 1F hypermethylation in the hippocampus of suicide victims with a history of childhood abuse, with methylation levels approximately 2-fold higher at key CpG sites than in controls. SKA2 hypomethylation in individuals who died by suicide.	55, 58, 59, 89, 90
Inflammatory markers	Elevated IL-6 reported in serum, plasma, CSF, and post-mortem prefrontal cortex of individuals with suicidal ideation (often pooled with attempts in the source studies). Elevated CRP, fibrinogen, and tryptophan catabolites (TRYCATs) reported in suicidal individuals.	Elevated CSF IL-6 highest in violent suicide attempters and correlates with attempt lethality. Elevated CRP, fibrinogen, and TRYCATs also reported (see ideation column). Reduced plasma omega-3 PUFAs (EPA, DHA) in MDD patients who later attempt suicide.	Significantly increased IL-1β, IL-6, and TNF-α at both mRNA and protein level in the prefrontal cortex (Brodmann area 10) of 24 teenage suicide victims compared with 24 matched controls.	64, 67, 69, 70, 74, 78
Genetic/GWAS	Polygenic risk scores for ADHD and autism spectrum disorder show positive associations with suicidal ideation in children and adolescents.	GWAS meta-analysis (Docherty et al. 2023) identified 12 genome-wide significant loci for suicide attempt across 43,871 cases (DRD2, SLC6A9, FURIN, NLGN1, SOX5, PDE4B, CACNG2). Polygenic risk scores for depressive symptoms, neuroticism, MDD, and schizophrenia predict suicide attempts.	Genetic studies specifically distinguishing death by suicide from suicide attempt are limited; most large-scale GWAS use suicide attempt as the phenotype.	38, 41, 43
Metabolic syndrome	UK Biobank cohort (*n* = 380,557) reported metabolic syndrome associated with increased suicidal ideation (OR 1.07, 95% CI 1.04–1.11).	Same UK Biobank cohort showed metabolic syndrome associated with increased risk of non-hospitalized attempt (OR 1.16) and hospitalized/fatal attempt (OR 1.60), with dose–response across number of components.	Same longitudinal cohort reported a combined endpoint of “suicide attempts resulting in hospitalization or death,” with dose-response across the number of metabolic syndrome components.	86

### Pediatric-specific patterns

A consistent thread across the domains reviewed in this manuscript is that pediatric biology cannot be treated as a smaller-scale version of adult biology. Adolescence is a developmental window of unique biological vulnerability, characterized by ongoing maturation of stress-response systems, prefrontal-limbic circuits, and reward processing pathways that are still calibrating their adult set-points (95). In this developmental context, several biomarkers reviewed here show patterns that diverge from, or even reverse, those documented in adult cohorts.

The clearest example comes from the HPA axis. Individuals with a history of suicide attempts show higher cortisol levels in samples with mean age below 40 years but lower cortisol in samples above 40 years (90). Rather than a quirk of age stratification, this reversal likely reflects a real developmental progression: the adolescent HPA axis is hyper-reactive to stressors, particularly social threat and peer rejection (95), and only later transitions to the blunted, exhausted profile characteristic of chronic adult HPA dysregulation. A similar developmental pattern emerges in the epigenetic regulation of the same axis. *Jokinen* et al. found that one CRH promoter CpG site (cg19035496) was hypomethylated in adults with violent suicide attempts but hypermethylated in adolescents at high psychiatric risk (58), indicating that the direction of stress-related methylation, not merely its magnitude, is age-dependent.

Neuroimaging findings follow a parallel trajectory. In the ABCD cohort, children aged 9–10 with suicidal thoughts and behaviors showed only minimal macroscopic structural differences (14), whereas adolescents aged 11–18 with a history of suicide attempts demonstrated reduced cortical surface area together with atypical thickening trajectories suggestive of delayed synaptic pruning (13).

Overall, these patterns argue against extrapolating adult biomarker reference ranges, mechanistic models, or treatment targets directly to pediatric populations. Developmentally-stratified study designs, age-specific biomarker calibration, and longitudinal cohorts spanning the pubertal transition are needed to clarify how the biology of suicidal ideation and attempt evolves across childhood and adolescence (95).

### Integrative framework

**Figure 1:**
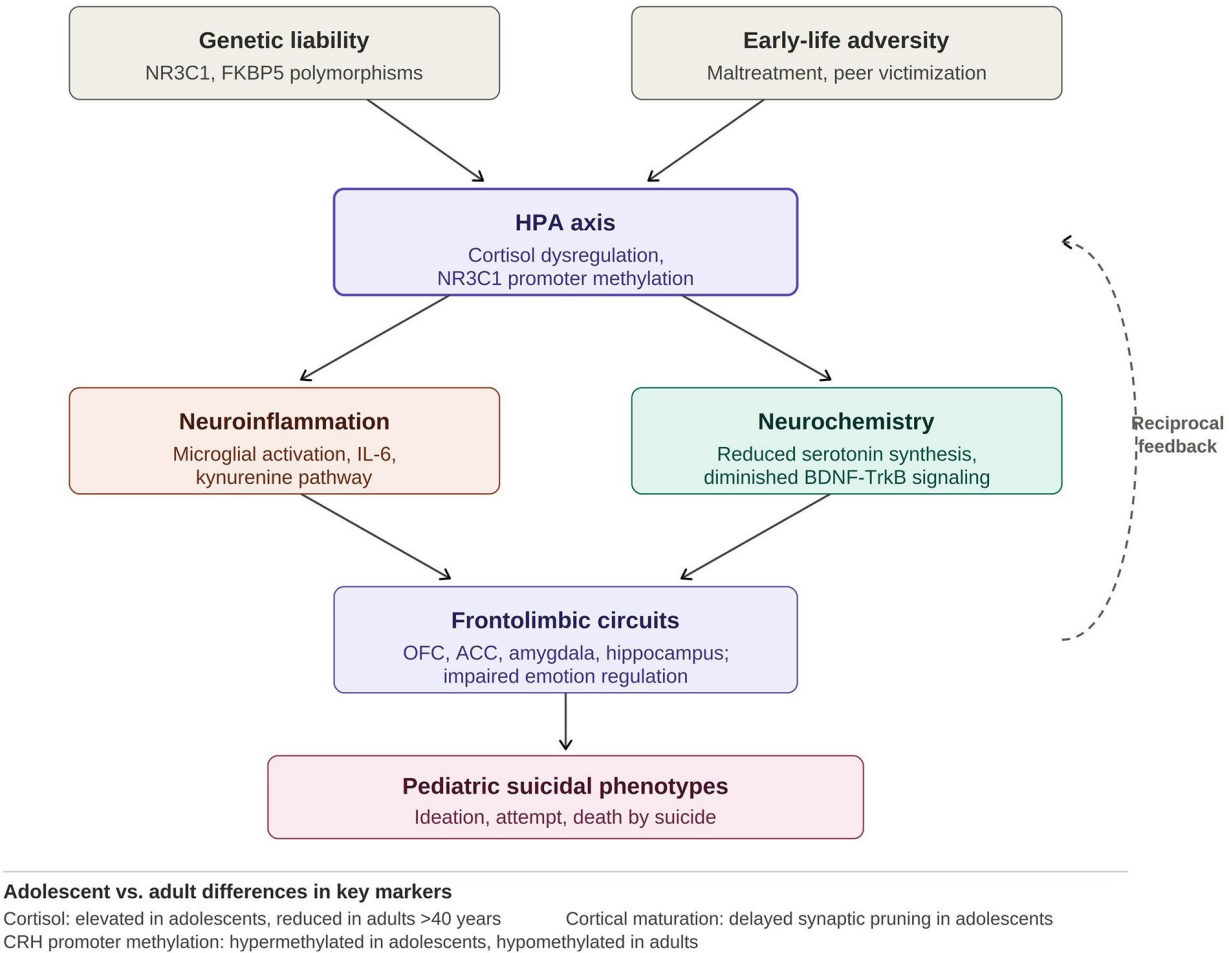
Integrative developmental cascade of pediatric suicidal vulnerability. Genetic liability (NR3C1 and FKBP5 polymorphisms) and early-life adversity converge on the hypothalamic-pituitary-adrenal (HPA) axis, which drives parallel downstream effects on neuroinflammation and neurochemistry. Both converge on frontolimbic circuits, whose impaired function underlies pediatric suicidal phenotypes. A reciprocal feedback loop sustains the cascade across development. Key markers reverse direction between adolescent and adult cohorts, as indicated in the lower panel.

The domains reviewed here can be understood as components of a single developmental cascade in which the HPA axis occupies a central position (Figure 1). Genetic liability and early-life adversity converge on glucocorticoid signaling. Polymorphisms in NR3C1 and FKBP5 alter glucocorticoid receptor sensitivity, while childhood maltreatment produces stable hypermethylation of the NR3C1 promoter that further reduces glucocorticoid feedback (55). Resulting cortisol dysregulation promotes microglial activation and elevated pro-inflammatory cytokines, particularly IL-6, which divert tryptophan metabolism toward neurotoxic kynurenine pathway products and away from serotonin synthesis (77). Reduced serotonergic tone, compounded by diminished BDNF–TrkB signaling, then compromises plasticity in the frontolimbic circuits whose alterations are the most reproducible neuroimaging finding in suicidal youth (11–13, 28). The cascade is also reciprocal. Inflammation amplifies HPA reactivity, and altered frontolimbic processing of social threat further drives stress-system activation, producing a positive feedback loop that may underlie the chronicity of suicidal vulnerability across the lifespan.

### Clinical implications and biomarker translation

No single biological marker is currently sufficient for individual-level risk prediction in pediatric suicidality, yet several findings carry plausible translational value. Peripheral markers with at least partially replicated pediatric and adolescent evidence, including IL-6, omega-3 polyunsaturated fatty acids, salivary or hair cortisol assessed with attention to circadian timing, and methylation at NR3C1 and SKA2, are best conceptualized as components of multimodal risk panels rather than as standalone indicators. Functional neuroimaging markers, particularly amplitude of low-frequency fluctuations in frontolimbic regions, have distinguished suicidal from non-suicidal depressed adolescents with over 80% sensitivity in single cohorts and may carry value in research-enriched clinical settings, although prospective replication remains a prerequisite for translation. Polygenic risk scores for depression and related psychiatric phenotypes are associated with suicide attempt at the group level but currently lack the individual-level discrimination required for clinical decision-making. A more tractable near-term translational goal is stratification rather than prediction, in which biologically defined subgroups of suicidal youth, such as those with elevated inflammatory tone, HPA hyperreactivity, or marked frontolimbic alterations, are identified for inclusion in mechanism-targeted trials. Whether such stratification will improve treatment response remains to be tested in pediatric populations.

### Future directions

Future pediatric studies should be designed with phenotype stratification built in from the outset, reporting separately on suicidal ideation, suicide attempt, and death by suicide rather than collapsing these outcomes. Longitudinal multimodal cohorts spanning the pubertal transition are needed to capture the developmental reversals in HPA, epigenetic, and neuroimaging signals identified throughout this review, and to integrate measures across biological domains within the same individuals. Methodological harmonization across cohorts, including shared sampling protocols, common assay platforms, and consensus phenotype definitions with standardized handling of non-suicidal self-injury, would improve comparability and enable future quantitative synthesis. Causal inference frameworks such as Mendelian randomization and target trial emulation are also required to move the field beyond cross-sectional association toward mechanistic and translational claims.

### Limitations

Several limitations should be acknowledged. Limitations of the underlying evidence base include: (i) the relative scarcity of direct pediatric data across all six biological domains reviewed, with much of the mechanistic literature still based on adult cohorts; (ii) the predominance of cross-sectional designs, which limit causal inference and conflate state and trait biology; (iii) heterogeneity in phenotype definition, biological sample type, assay platforms, and handling of medication exposure, all of which contribute to inconsistency across cohorts; (iv) the small number of multimodal studies integrating neuroimaging, genomics, epigenetics, and peripheral biomarkers in the same individuals; and (v) underrepresentation of racial, ethnic, and socioeconomic diversity in most large biobank-based cohorts, which constrains generalizability.

Limitations of the present review include its narrative design. We did not perform a PRISMA-compliant systematic search, formal risk-of-bias assessment, or quantitative meta-analysis. Restriction to English-language, peer-reviewed publications may have introduced language and publication bias. Finally, although priority was given to pediatric data, adult studies were included where pediatric evidence was lacking, and the relative weight of adult vs. pediatric evidence therefore varies across domains.

## Conclusion

**Figure 2:**
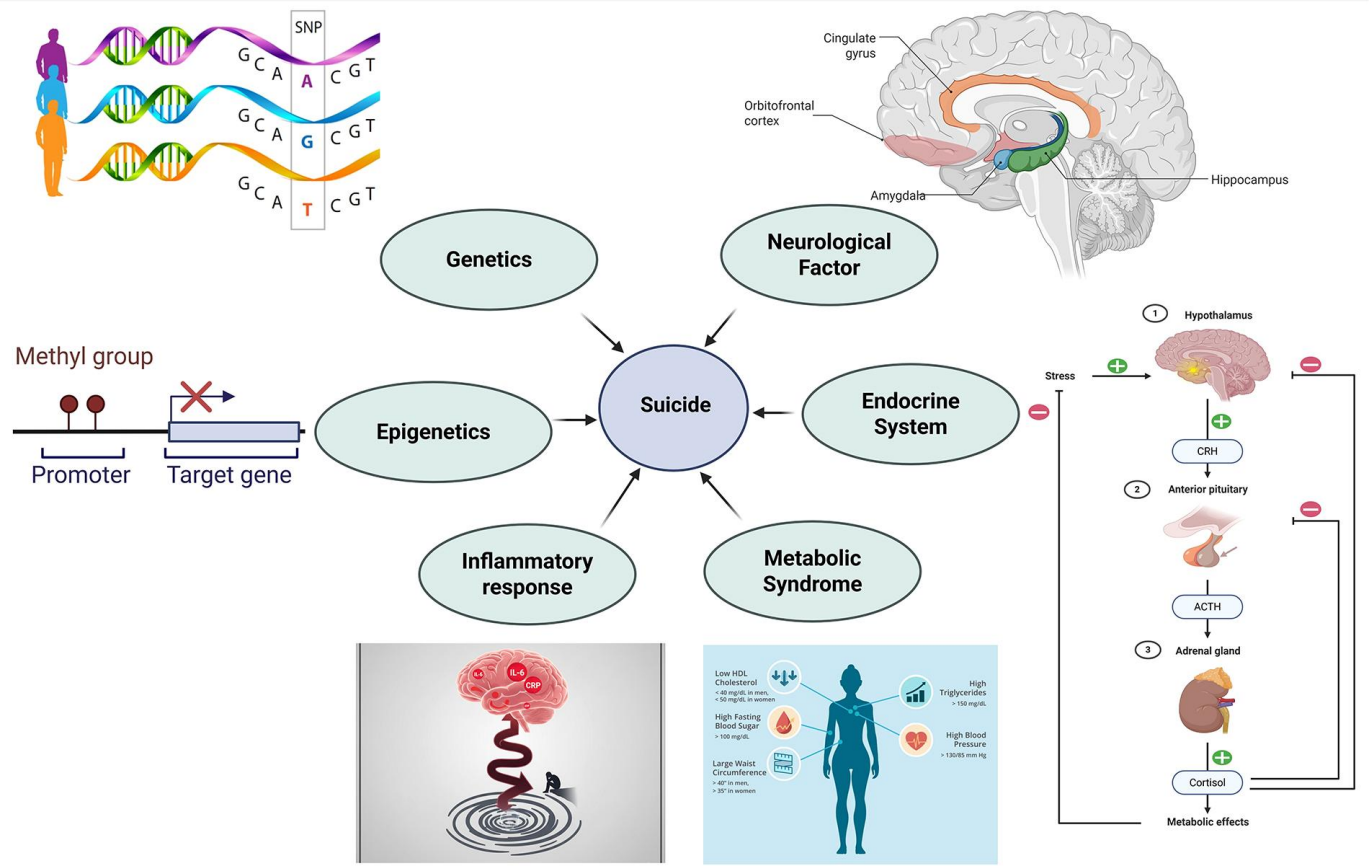
Multifactorial biological pathways implicated in suicide. Genetics, epigenetic modifications, neurological alterations including dysfunction in fronto-limbic regions, endocrine system dysregulation involving the hypothalamic–pituitary–adrenal axis, inflammatory responses such as elevated cytokines including IL-6 and C-reactive protein, and metabolic syndrome collectively contribute to suicide risk. These interconnected mechanisms highlight the complex biological underpinnings of suicidality.

Suicidal ideation, suicide attempt, and death by suicide in pediatric populations arise from interacting genetic, epigenetic, neurochemical, inflammatory, metabolic, and endocrine processes acting on a developing brain (Figure 2). The evidence reviewed suggests that suicidal ideation, suicide attempt, and death by suicide carry partially distinct biological signatures rather than representing a single continuum of severity. Several findings, including those related to cortisol regulation and stress-related DNA methylation, also differ in direction between pediatric and adult cohorts, underscoring that adult biomarker data cannot be directly extrapolated to youth. Advancing the field will require longitudinal, multimodal pediatric studies that disaggregate suicidal phenotypes, span the pubertal transition, and apply age-stratified reference ranges, with the longer-term goal of supporting biologically informed stratification and mechanism-targeted intervention.

## References

[1] Gaylor EM Stone DM Crosby AE. Suicide prevention: cDC’s approach to prevention. In: Rippe JM, editor. Lifestyle Medicine, Fourth Edition, Boca Raton: Taylor and Francis Group (2024). p. 1386–94.

[2] Pinheiro M Pais J Cabral T. Suicidal behavior in psychiatric population. Eur Psychiatry. (2015) 30:1. 10.1016/S0924-9338(15)31385-725169445

[3] Mirkovic B Laurent C Podlipski MA Frebourg T Cohen D Gerardin P. Genetic association studies of suicidal behavior: a review of the past 10 years, progress, limitations, and future directions. Front Psychiatry. (2016) 7:158. 10.3389/fpsyt.2016.00158 27721799 PMC5034008

[4] Centers for Disease Control and Prevention, National Center for Health Statistics. National Vital Statistics System, Mortality 2018–2022 on CDC WONDER Online Database, released 2024. Available from: Available online at: http://wonder.cdc.gov/mcd-icd10-expanded.html (Accessed September 9, 2024).

[5] Asarnow JR Porta G Spirito A Emslie G Clarke G Wagner KD Suicide attempts and nonsuicidal self-injury in the treatment of resistant depression in adolescents: findings from the TORDIA study. J Am Acad Child Adolesc Psychiatry. (2011) 50:772–81. 10.1016/j.jaac.2011.04.003 21784297 PMC3143365

[6] Edwards AC Ohlsson H Mościcki E Crump C Sundquist J Lichtenstein P On the genetic and environmental relationship between suicide attempt and death by suicide. Am J Psychiatry. (2021) 178:1060–9. 10.1176/appi.ajp.2020.20121705 34256608 PMC8570976

[7] Swanson S Eyllon M Sheu Y Miller M. Firearm access and adolescent suicide risk: toward a clearer understanding of effect size. Inj Prev. (2020) 27:264–70. 10.1136/injuryprev-2019-043605 32409621 PMC8165151

[8] Mann JJ Arango VA Avenevoli S Brent DA Champagne FA Clayton P Candidate endophenotypes for genetic studies of suicidal behavior. Biol Psychiatry. (2009) 65:556–63. 10.1016/j.biopsych.2008.11.021 19201395 PMC3271953

[9] Lengvenyte A Conejero I Courtet P Olié E. Biological bases of suicidal behaviours: a narrative review. Eur J Neurosci. (2021) 53:330–51. 10.1111/ejn.14635 31793103

[10] Arango V Underwood MD Boldrini M Tamir H Kassir SA Hsiung S Serotonin 1A receptors, serotonin transporter binding and serotonin transporter mRNA expression in the brainstem of depressed suicide victims. Neuropsychopharmacology. (2001) 25:892–903. 10.1016/s0893-133x(01)00310-4 11750182

[11] Schmaal L van Harmelen AL Chatzi V Lippard ETC Toenders YJ Averill LA Imaging suicidal thoughts and behaviors: a comprehensive review of 2 decades of neuroimaging studies. Mol Psychiatry. (2020) 25:408–27. 10.1038/s41380-019-0587-x 31787757 PMC6974434

[12] Johnston JAY Wang F Liu J Blond BN Wallace A Liu J Multimodal neuroimaging of frontolimbic structure and function associated with suicide attempts in adolescents and young adults with bipolar disorder. Am J Psychiatry. (2017) 174:667–75. 10.1176/appi.ajp.2016.15050652 28135845 PMC5939580

[13] Gifuni AJ Chakravarty MM Lepage M Ho TC Geoffroy MC Lacourse E Brain cortical and subcortical morphology in adolescents with depression and a history of suicide attempt. J Psychiatry Neurosci. (2021) 46:E347–57. 10.1503/jpn.200198 33961355 PMC8327980

[14] Vidal-Ribas P Janiri D Doucet GE Pornpattananangkul N Nielson DM Frangou S Multimodal neuroimaging of suicidal thoughts and behaviors in a U.S. Population-based sample of school-age children. Am J Psychiatry. (2021) 178:321–32. 10.1176/appi.ajp.2020.20020120 33472387 PMC8016742

[15] Cao J Chen X Chen J Ai M Gan Y Wang W Resting-state functional MRI of abnormal baseline brain activity in young depressed patients with and without suicidal behavior. J Affect Disord. (2016) 205:252–63. 10.1016/j.jad.2016.07.002 27467529

[16] Liu M Huang Y Li X Liu Y Yu R Long Y Aberrant frontolimbic circuit in female depressed adolescents with and without suicidal attempts: a resting-state functional magnetic resonance imaging study. Front Psychiatry. (2022) 13:1007144. 10.3389/fpsyt.2022.1007144 36386991 PMC9641155

[17] Zhang S Chen JM Kuang L Cao J Zhang H Ai M Association between abnormal default mode network activity and suicidality in depressed adolescents. BMC Psychiatry. (2016) 16:337. 10.1186/s12888-016-1047-7 27688124 PMC5041526

[18] Underwood MD Kassir SA Bakalian MJ Galfalvy H Dwork AJ Mann JJ Serotonin receptors and suicide, major depression, alcohol use disorder and reported early life adversity. Transl Psychiatry. (2018) 8:279. 10.1038/s41398-018-0309-1 30552318 PMC6294796

[19] Lester D. The concentration of neurotransmitter metabolites in the cerebrospinal fluid of suicidal individuals: a meta-analysis. Pharmacopsychiatry. (1995) 28:45–50. 10.1055/s-2007-979587 7542785

[20] Jankowski MP Sesack SR. Prefrontal cortical projections to the rat dorsal raphe nucleus: ultrastructural features and associations with serotonin and *γ*-aminobutyric acid neurons. J Comp Neurol. (2004) 468:518–29. 10.1002/cne.10976 14689484

[21] Sargin D Jeoung HS Goodfellow NM Lambe EK. Serotonin regulation of the prefrontal cortex: cognitive relevance and the impact of developmental perturbation. ACS Chem Neurosci. (2019) 10:3078–93. 10.1021/acschemneuro.9b00073 31259523

[22] Celada P Puig MV Artigas F. Serotonin modulation of cortical neurons and networks. Front Integr Neurosci. (2013) 7:25. 10.3389/fnint.2013.00025 23626526 PMC3630391

[23] Ninan PT van Kammen DP Scheinin M Linnoila M Bunney WE Goodwin FK. CSF 5-hydroxyindoleacetic Acid levels in suicidal schizophrenic patients. Am J Psychiatry. (1984) 141:566–9. 10.1176/ajp.141.4.566 6199986

[24] Sharma R Tikka SK Yadav A Bhute A Dhamija P Bastia B. Cerebrospinal fluid monoamine metabolite concentrations in suicide attempt: a meta-analysis. Asian J Psychiatr. (2021) 62:102711. 10.1016/j.ajp.2021.102711 34090249

[25] Mann JJ Huang YY Underwood MD Kassir SA Oppenheim S Kelly TM A serotonin transporter gene promoter polymorphism (5-HTTLPR) and prefrontal cortical binding in major depression and suicide. Arch Gen Psychiatry. (2000) 57:729–38. 10.1001/archpsyc.57.8.729 10920459

[26] Tyano S Zalsman G Ofek H Blum I Apter A Wolovik L Plasma serotonin levels and suicidal behavior in adolescents. Eur Neuropsychopharmacol. (2006) 16:49–57. 10.1016/j.euroneuro.2005.05.005 16076550

[27] Pfeffer CR McBride PA Anderson GM Kakuma T Fensterheim L Khait V. Peripheral serotonin measures in prepubertal psychiatric inpatients and normal children: associations with suicidal behavior and its risk factors. Biol Psychiatry. (1998) 44:568–77. 10.1016/S0006-3223(98)00020-1 9787881

[28] Pandey GN Ren X Rizavi HS Conley RR Roberts RC Dwivedi Y. Brain-derived neurotrophic factor and tyrosine kinase B receptor signalling in post-mortem brain of teenage suicide victims. Int J Neuropsychopharmacol. (2008) 11:1047–61. 10.1017/S1461145708009000 18611289

[29] Belujon P Grace AA. Dopamine system dysregulation in major depressive disorders. Int J Neuropsychopharmacol. (2017) 20:1036–46. 10.1093/ijnp/pyx056 29106542 PMC5716179

[30] Engström G Alling C Blennow K Regnéll G Träskman-Bendz L. Reduced cerebrospinal HVA concentrations and HVA/5-HIAA ratios in suicide attempters: monoamine metabolites in 120 suicide attempters and 47 controls. Eur Neuropsychopharmacol. (1999) 9:399–405. 10.1016/S0924-977X(99)00016-4 10523046

[31] Merali Z Du L Hrdina P Palkovits M Faludi G Poulter MO Dysregulation in the suicide brain: mRNA expression of corticotropin-releasing hormone receptors and GABAA receptor subunits in frontal cortical brain region. J Neurosci. (2004) 24:1478–85. 10.1523/JNEUROSCI.4734-03.2004 14960621 PMC6730322

[32] Peitl V Štefanović M Karlović D. Depressive symptoms in schizophrenia and dopamine and serotonin gene polymorphisms. Prog Neuropsychopharmacol Biol Psychiatry. (2017) 77:209–15. 10.1016/j.pnpbp.2017.04.011 28416295

[33] Arango V Huang YY Underwood MD Mann JJ. Genetics of the serotonergic system in suicidal behavior. J Psychiatr Res. (2003) 37:375–86. 10.1016/S0022-3956(03)00048-7 12849930

[34] Boldrini M Underwood MD Mann JJ Arango V. Serotonin-1A autoreceptor binding in the dorsal raphe nucleus of depressed suicides. J Psychiatr Res. (2008) 42:433–42. 10.1016/j.jpsychires.2007.05.004 17574270 PMC2268626

[35] Voracek M Loibl LM. Genetics of suicide: a systematic review of twin studies. Wien Klin Wochenschr. (2007) 119:463–75. 10.1007/s00508-007-0823-2 17721766

[36] Sokolowski M Wasserman J Wasserman D. Genome-wide association studies of suicidal behaviors: a review. Eur Neuropsychopharmacol. (2014) 24:1567–77. 10.1016/j.euroneuro.2014.08.006 25219938

[37] DiBlasi E Kang J Docherty AR. Genetic contributions to suicidal thoughts and behaviors. Psychol Med. (2021) 51:2148–55. 10.1017/S0033291721001720 34030748 PMC8477225

[38] Docherty AR Mullins N Ashley-Koch AE Qin X Coleman JR Shabalin A GWAS meta-analysis of suicide attempt: identification of 12 genome-wide significant loci and implication of genetic risks for specific health factors. Am J Psychiatry. (2023) 180:723–38. 10.1176/appi.ajp.21121266 37777856 PMC10603363

[39] Miranda A Shekhtman T McCarthy M DeModena A Leckband SG Kelsoe JR. Study of 45 candidate genes suggests CACNG2 may be associated with lithium response in bipolar disorder. J Affect Disord. (2019) 248:175–9. 10.1016/j.jad.2019.01.010 30738251 PMC7292366

[40] Li QS Shabalin AA DiBlasi E Gopal S Canuso CM Palotie A Genome-wide association study meta-analysis of suicide death and suicidal behavior. Mol Psychiatry. (2023) 28:891–900. 10.1038/s41380-022-01828-9 36253440 PMC9908547

[41] Ruderfer DM Walsh CG Aguirre MW Tanigawa Y Ribeiro JD Franklin JC Significant shared heritability underlies suicide attempt and clinically predicted probability of attempting suicide. Mol Psychiatry. (2020) 25:2422–30. 10.1038/s41380-018-0326-8 30610202 PMC6609505

[42] Lee D Baek JH Ha K Cho EY Choi Y Yang SY Dissecting the genetic architecture of suicide attempt and repeated attempts in Korean patients with bipolar disorder using polygenic risk scores. Int J Bipolar Disord. (2022) 10:3. 10.1186/s40345-022-00251-x 35112160 PMC8811109

[43] Lee PH Doyle AE Li X Silberstein M Jung JY Gollub RL Genetic association of attention-deficit/hyperactivity disorder and major depression with suicidal ideation and attempts in children: the adolescent brain cognitive development study. Biol Psychiatry. (2022) 92:236–45. 10.1016/j.biopsych.2021.11.026 35216811 PMC9213568

[44] Genis-Mendoza AD Hernández-Díaz Y González-Castro TB Tovilla-Zárate CA Castillo-Avila RG López-Narváez ML Association between TPH1 polymorphisms and the risk of suicide behavior: an updated meta-analysis of 18,398 individuals. Front Psychiatry. (2022) 13:932135. 10.3389/fpsyt.2022.932135 35928776 PMC9343722

[45] Fanelli G Serretti A. The influence of the serotonin transporter gene 5-HTTLPR polymorphism on suicidal behaviors: a meta-analysis. Prog Neuropsychopharmacol Biol Psychiatry. (2019) 88:375–87. 10.1016/j.pnpbp.2018.08.007 30125622

[46] Hernández-Díaz Y Genis-Mendoza AD González-Castro TB Tovilla-Zárate CA Juárez-Rojop IE López-Narváez ML Association and genetic expression between genes involved in HPA axis and suicide behavior: a systematic review. Genes (Basel). (2021) 12:1608. 10.3390/genes12101608 34681002 PMC8536196

[47] Sanabrais-Jiménez MA Esquivel-López AA Sotelo-Ramírez CE Aguilar-García A Ordoñez-Martínez B Jiménez-Pavón J NR3C1 And NR3C2 genes increase the risk of suicide attempt in psychiatric disorder patients with history of childhood trauma. Neuropsychiatr Dis Treat. (2023) 19:2561–71. 10.2147/NDT.S431176 38035135 PMC10683665

[48] Peng Q Yan H Wen Y Lai C Shi L. Association between NR3C1 rs41423247 polymorphism and depression: a PRISMA-compliant meta-analysis. Medicine (Baltimore). (2018) 97:e12541. 10.1097/MD.0000000000012541 30278546 PMC6181539

[49] Castro-Vale I Durães C van Rossum EFC Staufenbiel SM Severo M Lemos MC The glucocorticoid receptor gene (NR3C1) 9*β* SNP is associated with posttraumatic stress disorder. Healthcare (Basel). (2021) 9:173. 10.3390/healthcare9020173 33562675 PMC7915937

[50] Hawn SE Sheerin CM Lind MJ Hicks TA Marraccini ME Bountress K Gxe effects of FKBP5 and traumatic life events on PTSD: a meta-analysis. J Affect Disord. (2019) 243:455–62. 10.1016/j.jad.2018.09.058 30273884 PMC6487483

[51] Caspi A Sugden K Moffitt TE Taylor A Craig IW Harrington H Influence of life stress on depression: moderation by a polymorphism in the 5-HTT gene. Science. (2003) 301:386–9. 10.1126/science.1083968 12869766

[52] Antypa N Serretti A Rujescu D. Serotonergic genes and suicide: a systematic review. Eur Neuropsychopharmacol. (2013) 23:1125–42. 10.1016/j.euroneuro.2013.03.013 23742855

[53] Palma-Gudiel H Fañanás L. An integrative review of methylation at the serotonin transporter gene and its dialogue with environmental risk factors, psychopathology and 5-HTTLPR. Neurosci Biobehav Rev. (2017) 72:190–209. 10.1016/j.neubiorev.2016.11.011 27880876

[54] Lim DH Maher ER. DNA Methylation: a form of epigenetic control of gene expression. Obstet Gynaecol. (2010) 12:37–42. 10.1576/toag.12.1.037.27556

[55] McGowan PO Sasaki A D'Alessio AC Dymov S Labonté B Szyf M Epigenetic regulation of the glucocorticoid receptor in human brain associates with childhood abuse. Nat Neurosci. (2009) 12:342–8. 10.1038/nn.2270 19234457 PMC2944040

[56] Labonté B Suderman M Maussion G Navaro L Yerko V Mahar I Genome-wide epigenetic regulation by early-life trauma. Arch Gen Psychiatry. (2012) 69:722–31. 10.1001/archgenpsychiatry.2011.2287 22752237 PMC4991944

[57] Bustamante AC Aiello AE Galea S Ratanatharathorn A Noronha C Wildman DE Glucocorticoid receptor DNA methylation, childhood maltreatment and major depression. J Affect Disord. (2016) 206:181–8. 10.1016/j.jad.2016.07.038 27475889 PMC5077661

[58] Jokinen J Boström AE Dadfar A Ciuculete DM Chatzittofis A Åsberg M Epigenetic changes in the CRH gene are related to severity of suicide attempt and a general psychiatric risk score in adolescents. EBioMedicine. (2018) 27:123–33. 10.1016/j.ebiom.2017.12.018 29277323 PMC5828554

[59] Guintivano J Brown T Newcomer A Jones M Cox O Maher BS Identification and replication of a combined epigenetic and genetic biomarker predicting suicide and suicidal behaviors. Am J Psychiatry. (2014) 171:1287–96. 10.1176/appi.ajp.2014.14010008 25073599 PMC7081376

[60] Kang HJ Kim JM Lee JY Kim SY Bae KY Kim SW BDNF Promoter methylation and suicidal behavior in depressive patients. J Affect Disord. (2013) 151:679–85. 10.1016/j.jad.2013.08.001 23992681

[61] Koning SM Kessler CL Canli T Duman EA Adam EK Zinbarg R Early-life adversity severity, timing, and context type are associated with SLC6A4 methylation in emerging adults: results from a prospective cohort study. Psychoneuroendocrinology. (2024) 170:107181. 10.1016/j.psyneuen.2024.107181 39298801 PMC13741894

[62] Bani-Fatemi A Jeremian R Wang KZ Silveira J Zai C Kolla NJ Epigenome-wide association study of suicide attempt in schizophrenia. J Psychiatr Res. (2018) 104:192–7. 10.1016/j.jpsychires.2018.07.011 30103066

[63] Perret LC Geoffroy MC Barr E Parnet F Provencal N Boivin M Associations between epigenetic aging and childhood peer victimization, depression, and suicidal ideation in adolescence and adulthood: a study of two population-based samples. Front Cell Dev Biol. (2023) 10:1051556. 10.3389/fcell.2022.1051556 36712964 PMC9879289

[64] Martinez JM Garakani A Yehuda R Gorman JM. Proinflammatory and “resiliency” proteins in the CSF of patients with major depression. Depress Anxiety. (2012) 29:32–8. 10.1002/da.20876 21898706

[65] Karlović D Serretti A Vrkić N Martinac M Marčinko D. Serum concentrations of CRP, IL-6, TNF-α and cortisol in major depressive disorder with melancholic or atypical features. Psychiatry Res. (2012) 198:74–80. 10.1016/j.psychres.2011.12.007 22386567

[66] Hoyo-Becerra C Huebener A Trippler M Lutterbeck M Liu ZJ Truebner K Concomitant interferon alpha stimulation and TLR3 activation induces neuronal expression of depression-related genes that are elevated in the brain of suicidal persons. PLoS One. (2013) 8:e83149. 10.1371/journal.pone.0083149 24391741 PMC3877033

[67] Pandey GN Rizavi HS Ren X Fareed J Hoppensteadt DA Roberts RC Proinflammatory cytokines in the prefrontal cortex of teenage suicide victims. J Psychiatr Res. (2012) 46:57–63. 10.1016/j.jpsychires.2011.08.006 21906753 PMC3224201

[68] González-Castro TB Tovilla-Zárate CA López-Narváez ML Genis-Mendoza AD Juárez-Rojop IE. Interleukin-6 levels in serum, plasma, and cerebral spinal fluid in individuals with suicide behavior: systematic review and meta-analysis with meta-regression. J Interferon Cytokine Res. (2021) 41:258–67. 10.1089/jir.2020.0265 34280025

[69] Lindqvist D Janelidze S Hagell P Erhardt S Samuelsson M Minthon L Interleukin-6 is elevated in the cerebrospinal fluid of suicide attempters and related to symptom severity. Biol Psychiatry. (2009) 66:287–92. 10.1016/j.biopsych.2009.01.030 19268915

[70] Shah IR Manzoor S Hussain A Majid S Khan K. Association of interleukin-6 (IL-6) with suicide attempt. Ind Psychiatry J. (2025) 34:433–7. 10.4103/ipj.ipj_77_25 41180063 PMC12574729

[71] Ganança L Oquendo MA Tyrka AR Cisneros-Trujillo S Mann JJ Sublette ME. The role of cytokines in the pathophysiology of suicidal behavior. Psychoneuroendocrinology. (2016) 63:296–310. 10.1016/j.psyneuen.2015.10.008 26546783 PMC4910882

[72] Kim YK Lee SW Kim SH Shim SH Han SW Choi SH Differences in cytokines between non-suicidal patients and suicidal patients in major depression. Prog Neuropsychopharmacol Biol Psychiatry. (2008) 32:356–61. 10.1016/j.pnpbp.2007.08.041 17919797

[73] Janelidze S Suchankova P Ekman A Erhardt S Sellgren C Samuelsson M Low IL-8 is associated with anxiety in suicidal patients: genetic variation and decreased protein levels. Acta Psychiatr Scand. (2015) 131:269–78. 10.1111/acps.12339 25251027

[74] Fatemian H Moslemi H Hosseini Y Moshfeghinia R. C-reactive protein (CRP) level in depressed patients with suicidal behavior: a systematic review and meta-analysis. J Affect Disord. (2024) 366:423–33. 10.1016/j.jad.2024.08.135 39187188

[75] Patel S Govindarajan V Chakravarty S Dubey N. From blood to brain: exploring the role of fibrinogen in the pathophysiology of depression and other neurological disorders. Int Immunopharmacol. (2024) 143:113326. 10.1016/j.intimp.2024.113326 39388892

[76] Almulla AF Thipakorn Y Vasupanrajit A Tunvirachaisakul C Oxenkrug G Al-Hakeim HK The tryptophan catabolite or kynurenine pathway in a major depressive episode with melancholia, psychotic features and suicidal behaviors: a systematic review and meta-analysis. Cells. (2022) 11:3112. 10.3390/cells11193112 36231075 PMC9563030

[77] Stone TW Perkins MN. Quinolinic acid: a potent endogenous excitant at amino acid receptors in CNS. Eur J Pharmacol. (1981) 72:411–2. 10.1016/0014-2999(81)90587-2 6268428

[78] Plana T Gracia R Méndez I Pintor L Lazaro L Castro-Fornieles J. Total serum cholesterol levels and suicide attempts in child and adolescent psychiatric inpatients. Eur Child Adolesc Psychiatry. (2010) 19:615–9. 10.1007/s00787-009-0084-x 20047063

[79] Cho H Guo G Iritani BJ Hallfors DD. Genetic contribution to suicidal behaviors and associated risk factors among adolescents in the U.S. Prev Sci. (2006) 7:303–11. 10.1007/s11121-006-0042-5 16775759

[80] Schiweck C Claes S Van Oudenhove L Lafit G Vaessen T de Beeck GO Childhood trauma, suicide risk and inflammatory phenotypes of depression: insights from monocyte gene expression. Transl Psychiatry. (2020) 10:296. 10.1038/s41398-020-00979-z 32839428 PMC7445278

[81] Howe SJ Hewitt K Baraskewich J Cassidy S McMorris CA. Suicidality among children and youth with and without autism spectrum disorder: a systematic review of existing risk assessment tools. J Autism Dev Disord. (2020) 50:3462–76. 10.1007/s10803-020-04394-7 32100237

[82] Nusslock R Miller GE. Early-life adversity and physical and emotional health across the lifespan: a neuroimmune network hypothesis. Biol Psychiatry. (2016) 80:23–32. 10.1016/j.biopsych.2015.05.017 26166230 PMC4670279

[83] Chang JC Yen AM Lee CS Chen SL Chiu SY Fann JC Metabolic syndrome and the risk of suicide: a community-based integrated screening samples cohort study. Psychosom Med. (2013) 75:807–14. 10.1097/PSY.0000000000000014 24163389

[84] Moreira FP Jansen K Cardoso TD Mondin TC Magalhaes PV Kapczinski F Metabolic syndrome and psychiatric disorders: a population-based study. Braz J Psychiatry. (2018) 41:38–43. 10.1590/1516-4446-2017-2328 30328961 PMC6781708

[85] Liu Z Sun L Sun F Zhang Y Wang J Zhang Z The abnormalities of lipid metabolism in children and adolescents with major depressive disorder and relationship with suicidal ideation and attempted suicide. Heliyon. (2024) 10:e29198. 10.1016/j.heliyon.2024.e30344 38726112 PMC11079100

[86] Zhao Z Xie M Tao S Lv Q Zhang J Cai J Metabolic syndrome increases the risk of suicide attempt: evidence from a population-based cohort and genomic analysis. Transl Psychiatry. (2025) 15:365. 10.1038/s41398-025-03575-1 41052968 PMC12501300

[87] Penninx BW Milaneschi Y Lamers F Vogelzangs N. Understanding the somatic consequences of depression: biological mechanisms and the role of depression symptom profile. BMC Med. (2013) 11:129. 10.1186/1741-7015-11-129 23672628 PMC3661358

[88] Muldoon MF Mackey RH Korytkowski MT Flory JD Pollock BG Manuck SB. The metabolic syndrome is associated with reduced central serotonergic responsivity in healthy community volunteers. J Clin Endocrinol Metab. (2006) 91:718–21. 10.1210/jc.2005-1654 16303834

[89] Hernández-Díaz Y González-Castro TB Tovilla-Zárate CA Juárez-Rojop IE López-Narváez ML Pérez-Hernández N The role of peripheral cortisol levels in suicide behavior: a systematic review and meta-analysis of 30 studies. Psychiatry Res. (2020) 293:113448. 10.1016/j.psychres.2020.113448 32971405

[90] O'Connor DB Ferguson E Green JA O'Carroll RE O'Connor RC. Cortisol levels and suicidal behavior: a meta-analysis. Psychoneuroendocrinology. (2016) 63:370–9. 10.1016/j.psyneuen.2015.10.011 26555430

[91] González-Castro TB Almeida de la OPD Tovilla-Zárate CA López-Narváez ML Genis-Mendoza AD Juárez-Rojop IE Evaluation of leptin levels in serum as a biomarker for suicide behavior: systematic review and meta-analysis. Int J Neurosci. (2021) 131:49–55. 10.1080/00207454.2020.1733558 32083967

[92] Acikel SB Eroglu C Ugras Dikmen A Kurar E. The association between leptin receptor polymorphism and suicidal behaviour in depressed adolescents. Int J Psychiatry Clin Pract. (2020) 24:120–6. 10.1080/13651501.2019.1711422 31916884

[93] Fu XL Li X Ji JM Wu H Chen HL. Blood hormones and suicidal behaviour: a systematic review and meta-analysis. Neurosci Biobehav Rev. (2022) 139:104725. 10.1016/j.neubiorev.2022.104725 35690122

[94] Tymofiyeva O Reeves KW Shaw C Lopez E Aziz S Max JE A systematic review of MRI studies and the “emotional paiN and social disconnect (END)”. brain Model of Suicidal Behavior in Youth. Behav Neurol. (2023) 2023:7254574. 10.1155/2023/7254574 37786433 PMC10541999

[95] Miller AB Prinstein MJ. Adolescent suicide as a failure of acute stress-response systems. Annu Rev Clin Psychol. (2019) 15:425–50. 10.1146/annurev-clinpsy-050718-095625 30786243 PMC6953613

